# Tobacco smoke exposure as a risk factor for human papillomavirus infections in women 18-26 years old in the United States

**DOI:** 10.1371/journal.pone.0223532

**Published:** 2019-10-30

**Authors:** Philip Kum-Nji, Linda Meloy, Lori Keyser-Marcus

**Affiliations:** 1 Children’s Hospital of Richmond at the Virginia Commonwealth University School of Medicine, Richmond, Virginia, United States of America; 2 Department of Psychiatry, Virginia Commonwealth University, Richmond, Virginia, United States of America; Universidad Miguel Hernandez de Elche, SPAIN

## Abstract

**Background:**

Although tobacco smoke has been associated with many infections, little is known of its association with human papillomavirus (HPV) infections among young adult women. The aim of the study was to explore the association of tobacco smoke exposure on HPV infections in young adult women in the United States. It was hypothesized that tobacco smoke exposure (both active and passive) as objectively measured by cotinine levels was associated with increased HPV infection in a national sample of 18 and 26 year-old women in the United States.

**Study methods and findings:**

The NHANES 2007–2012 dataset was used in the analyses. A national representative sample of women 18 to 26 year old (N = 1,414) was included in the study. Infection with any HPV was determined by PCR while tobacco smoke exposure was determined by measuring serum cotinine levels. Women with cotinine levels <0.05 ng/mL were considered unexposed and those with levels > = 0.05 were considered as exposed. Exposed women were further categorized as passive smokers (cotinine levels 0.05-<10 ng/mL, while active smokers were those with cotinine levels = > 10ng/mL). Data were analyzed by descriptive statistics and multiple logistic regression analysis. Exposed women (passive smokers with cotinine levels > = 0.05ng/mL-10ng/mL) were almost 2 times (64% vs 35%) more likely to be infected with any HPV type than their unexposed counterparts (cotinine levels <0.05ng/mL). Also women who were active smokers (cotinine levels > = 10 ng/mL) were more than twice more likely (75%) to be infected with the virus than the unexposed counterparts. The relationship held true even after controlling for various socio-demographics. Indeed in the multiple regression analyses controlling for the various confounders, compared to their unexposed counterparts, women exposed to second hand smoke were more than twice more likely to have HPV infections (OR: 2.45; 95% C.I = 1.34–4.48). When compared to their unexposed counterparts, actively smoking women were more than 3.5 times more likely to be infected with HPV (OR = 3.56; 95% CI 1.23–10.30).

Finally, a strong dose-response relationship was further demonstrated with increasing risk of HPV with each quartile of cotinine levels even after controlling for various confounders. The risk of HPV was lowest in the lowest quartile (Referent OR = 1) and increased steadily with each quartile of cotinine levels until the risk was highest among women with cotinine levels in the 4^th^ quartile (OR = 4.16; 95% C.I. = 1.36–12.67).

**Conclusion:**

Both passive and active tobacco smoking were strongly associated with any HPV infection in 18 to 26 year old young women with a significant dose-response relationship. Future studies should explore the effect of tobacco smoke exposure among younger women less than 18 years of age.

## Introduction

Human papillomavirus (HPV) is probably the most common sexually transmitted infection in the United States affecting nearly all sexually active men and women. About 80 million Americans may be currently infected with the virus with almost 14 million new cases annually [1]. More than 150 types of HPV are known and about 40 types are sexually transmitted and can infect the anogenital and other mucosal sites. It has been shown that 66% of all cervical cancers, 55% of vaginal cancers, 79% of anal cancers, and 62% of oropharyngeal cancers are attributable to high-risk HPV (e.g. types 16 and 18) while low-risk HPV (e.g. types 6 and 11) cause ano-genital, oropharyngeal, and respiratory papillomatosis [2,3].

Known risk factors include, early initiation of sexual intercourse, frequency of sexual intercourse especially with multiple partners, ethnicity, and age [1–4]. Although tobacco smoke exposure has been significantly associated with many infectious illnesses [5], only a few studies in the literature show that active smoking and environmental tobacco smoke exposure (ETS) are risk factors for HPV infections [4,6,7]. However, these studies determined the smoking status of the subjects by history alone and without the use of any biomarkers.

The present study was aimed at determining the strength of the association of tobacco smoke exposure on HPV infections among a representative sample of young adult US women 18–26 years.

## Methods

### Design and study population

The NHANES database was used for our analysis. The NHANES is a comprehensive research assessment of health and nutritional status of adults and children in the United States, which utilizes data collected through both interview and physical exam findings including medical, dental, and laboratory tests [8].

NHANES data is and ongoing collection of data nationwide representative sample using stratified cluster complex sampling techniques by the CDC. Some population groups are over sampled in order to obtain accurate information on these groups not previously well studied. Full details of the complex sampling procedures have been described elsewhere [8].

Subjects are questioned to obtain detailed socio-demographic information of all household members. In addition, subjects are asked to provide blood samples (in mobile examination centers (MEC), to assess serum cotinine levels. Serum cotinine levels were measured by an isotope dilution-high performance liquid chromatography [9]. Data from 2007 to 2012 were merged for the present analysis. Data merged were demographic data, serum cotinine levels, and HPV infections as determined by vaginal swab PCR. Data for 1,414 women 18 and 26 years of age were abstracted for analysis. Vaginal swab HPV PCRs were not available for younger age groups. The IRB of the Virginia Commonwealth University approved the study as exempt.

### Definition of terms

Tobacco smoke was classified into three levels. Subjects with serum cotinine levels <0.05 were defined as unexposed and non-smokers; individuals with levels of 0.05–10ng/mL were defined as exposed to second hand smoke, while those with levels > = 10 ng/mL were classified as active smokers. To further determine the effect of increasing cotinine levels on HPV infections, cotinine levels were categorized into 4 groups (quartiles).

HPV infection was determine by the method of Roche Linear Array PCR using vaginal swabs and able to detect 37 HPV types viz 6,11,16,18,26,31,33,35,39,40,42,45,51,52,53,54,55,56,58,59,61,62,64,66,67,68,69,70,71,72,73,81,82,83,84,89,and 1539 [8, 9]. Age of women was classified into 2 groups of late teens and early adulthood: <20 years and 20+ years. Income was classified into 2 categories of < $55,000 and $55,000+ since this was the median income for the sample during the study period.

For the purpose of this analysis, race of the women was categorized as white and non-white.

Average age of sex initiation was 16 years. The standard deviation of age at sex initiation was ~2 years. Therefore we dichotomized this variable into <14 years and 14+ years. The median number of lifetime sex partners was 4, with a mode of 1 so we dichotomized this variable into < 2 and 2 + for the analysis.

### Statistical analysis

The outcome variable of interest was HPV infection among 18 to 26year old women. The primary independent variable of interest was tobacco smoke exposure as objectively measured by cotinine levels.

Four previously identified socio-demographic variables associated with HPV infections included to control for confounding were age of woman, race/ethnicity, life-time sexual partners, and age at sexual initiation. One potential confounder not previously explored was income.

Descriptive statistics using X2 test for categorical variables were conducted to compare the proportions of subjects with HPV infections by selected socio-demographic variables. Multiple logistic regression analysis was further conducted to determine whether tobacco smoke exposure was still predictive of HPV infection after controlling for the other confounding variables. Because of the skewedness of cotinine levels, we further explored the impact of each quartile of cotinine level on HPV infection in order to determine if there was any dose-response relationship. A p-value of <0.05 was used as a test of significance for all cases. Because some population subgroups were oversampled to obtain unbiased estimates representative of US population, analyses were performed using the IBM SPSS complex sample analyses software for Windows, Version 240, Armonk, NY [10].

## Results

### Socio-demographics by HPV status

Table 1 shows the socio-demographic characteristics of the study population in relation to HPV infection. Number of lifetime male sex partners was strongly associated with HPV infections (p <0.001). Overall, any tobacco smoke exposure i.e. exposed vs. no exposure (as measured by cotinine levels) were strongly associated with HPV infections (p<0.001). Even when tobacco smoke exposure was categorized into three groups of unexposed (serum cotinine levels <0.05ng/mL), second hand smoke (cotinine levels 0.05-<10 ng/mL), and active smoking (cotinine levels 10+), tobacco smoke exposure was still strongly predictive of HPV infections suggesting a dose-response relationship. Because cotinine levels are skewed to the right, tobacco smoke exposure (by cotinine levels) was further categorized into 4 ascending groups (quartiles) in order to further to test for a dose-response relationship with HPV infections. The prevalence of HPV infection increased steadily from the first quartile to the 4^th^ quartile of cotinine levels with a strong dose-response relationship (p <0.001).

**Table 1:** Prevalence of HPV infections by selected socio-demographic characteristics among women 18–26 years in the United States.

% HPV Infection by PCR (N = 1234)		
Variable	Weighted % with HPV (95%CI)	P Value
All (N = 1234)	54.6 (48.8–60.2)	-
Lifetime Number of Male Sexual Partners (N = 1101)		
0–1 partner	17.8 (10.5–28.5)	
2 or more partners	67.0 (61.4–72.2)	<0.001
Age at Sexual Initiation in years (N = 1061)		
<14	74.9 (59.3–86.0)	
14+	57.3 (51.7–62.8)	0.030
Race (N = 1234)		
White	50.3 (42.3–58.4)	
Non- White	60.3 (54.8–65.5)	0.012
Annual Household Income (N = 1124)		
< $55,000	60.4 (52.5–67.8)	
$55,000+	41.7 (30.2–54.2)	0.027
Age of Woman in Years (N = 1234)		
<20	54.0 (39.0–68.4)	
20+	54.6 (48.6–60.5	0.940
Tobacco Smoke Exposure by Cotinine Levels (ng/mL; N = 1172)		
Unexposed (<0.05)	35.9 (28.3–44.3)	
Second Hand Smoke (0.05–10)	63.7 (54.8–71.7)	
Active Smoking (10+)	74.1 (59.7–84.7)	<0.001
Tobacco Smoke Exposure (cotinine Quartiles in ng/mL; N = 1172)		
<0.021	35.8 (26.4–46.5)	
0.021–0.116	46.4 (38.0–55.0)	
0.116–3.420	63.1 (53.2–72.0)	
3.420+	74.8 (61.0–84.9)	<0.001

The other variables associated with HPV infections were annual household income (p = 0.021), race (p = 0.022), and age at sexual initiation (p = 0.030). Poorer women (yearly household income < $55,000 were more likely to be infected than their higher income counterparts. Women who initiated sexual intercourse with men earlier than 14 years were more likely to be infected than those who initiated sex at a later age.

### Prevalence of HPV infections and tobacco smoke exposure among 18 and 26 year old women

Of the 1414 women included in the study, cotinine levels were available for 1309 women. Of these, at least 60% were either exposed to tobacco smoke or were active smokers (cotinine levels >0.05ng/mL), while only 40% were unexposed (cotinine levels <0.05ng/mL i.e. not exposed and not actively smoking.

**Fig 1:**
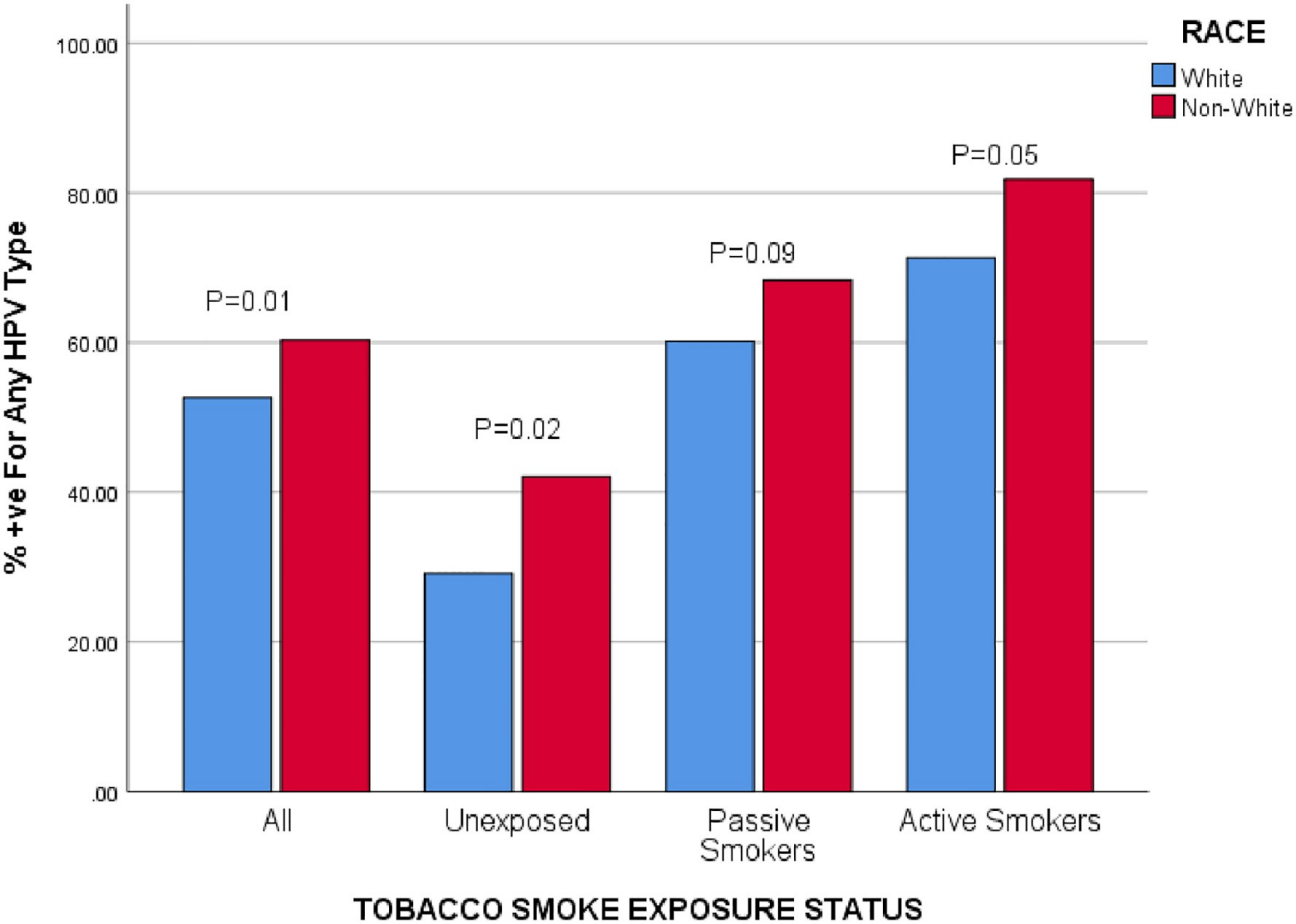
Prevalence of HPV infections by tobacco smoke exposure status and by race. The above Figure shows that overall, regardless of exposure status to tobacco smoke, none-white women 18–26 years were more likely to be infected with any HPV type than their white counterparts (P = 0.01). Even when exposure status was considered, similar differences were more or less noted for unexposed women (serum cotinine levels <0.05ng/mL, P = 0.02). Racial differences were less significant among passive smokers (serum cotinine levels (0.05–10ng/mL, P = 0.09) than among active smokers (serum cotinine levels 10+ ng/mL, P = 0.05).

Vaginal swab PCRs were available for only 1234 women. About 55% these women were infected with at least one of the 37 HPV types. Almost 40% of all women had oncogenic type HPV (also see Table 2). Overall, HPV infections were more likely to occur among Non-White than White women (P = 0.01). Non-White women had persistently higher HPV infections among the 3 categories of tobacco smoke exposure although the difference was more significant among the unexposed women (P = 0.02), followed by the actively smoking group (P = 0.05) and least among the passive smoking group (P = 0.09; also see Fig 1).

**Table 2:** Prevalence of HPV infections and tobacco smoke exposure in women 18–26 years.

% HPV Infection by PCR (N = 1234)	Weighted % (95% CI)
Yes (Any)	54.6 (48.8–60.2)
No	45.4 (39.8–51.2)
Oncogenic only*	37.2 (32.6–42.1)
% Tobacco Smoke Exposure by Cotinine Levels (N = 1309)	Weighted % (95% CI)
Unexposed (0.05ng/mL)	39.3 (34.7–44.2)
Second Hand Smoke (0.05–10ng/mL)	39.7 (34.1–45.6)
Active Smoking (10+ ng/mL)	20.9 (16.1–26.7)

*13 oncogenic (high-risk) HPV types in this study: 16, 18,31,33,35,39,45,51,52,56,58,66,69.

24 non-oncogenic (low-risk) HPV types: 6,11,26,40,42,53,54,55,59,61,62,64,67,68,70,71,72,73,81,82,83,84,89,and 1539.

### Multiple regression analysis of factors associated with HPV infections among 18 and 26 year old women

In a multiple logistic regression analysis (see Table 3), number of life-time sex partners was by far the single most significant predictor of HPV infection. The risk of HPV infection was over 9 times higher among those with at least 2 lifetime male sex partner as compared to 2+ (OR = 9.08; CI = 4.05–20.37). Even after controlling for other confounders, compared to unexposed subjects (cotinine levels <0.05ng/mL), women with serum cotinine levels in the exposed range (0.05–10ng/mL) were more than 2 times more likely to be infected while those who were active smokers (cotinine levels >10ng/mL) were more than 3 times more likely to be infected with HPV. A dose-repose relationship was thus demonstrated. To further demonstrate the importance of this dose-response relationship, cotinine levels were divided into quartiles, because of the general skewedness of cotinine levels. Tobacco smoke exposure was still strongly predictive of HPV infections. Thus HPV infection risk steadily increased with each quartile of cotinine levels with lowest risk in unexposed group (referent) to the highest risk in the last quartile (OR = 4.2; 95% CI = 1.4–12.7; also see Table 4).

**Table 3:** Weighted logistic regression of the impact of tobacco smoke exposure on HPV infections: Adjusted ORs and their 95% CIs.

Variable	Adjusted OR (95% CI)
No Sex Male Sex Partners (2+ Vs < 2)	9.08 (4.05–20.37)
Exposure Status	
Unexposed (Referent)	1
Passive Smoke (vs. Unexposed)	2.45 (1.34–4.48)
Active Smoking (vs. Unexposed)	3.56(1.23–10.30)
Race/Ethnicity (Non-White Vs White	1.76 (1.06–2.92)
Age at Sexual Initiation in years (< 14 vs 14+)	1.05 (0.40–2.76)
Age of Young Adult Woman in years (20+ Vs <20)	1.52 (0.67–3.48)
Household Income (<55,000 Vs 55,000+)	1.62 (0.78–3.36)

**Table 4:** Linear trend showing the impact of quartiles of cotinine levels on HPV infections in young adult women in the United States*.

Quartiles of Cotinine Levels	Adjusted OR (95% CI)
1^st^ (Referent)	1
2^nd^ vs 1^st^	1.40 (0.77–2.81)
3^rd^ vs 1^st^	2.12 (1.08–4.13)
4^th^ vs 1^st^	4.16 (1.36–12.67)

*Adjusted for Lifetime sex partners, age at sex initiation, age of woman, Race, and family household income level (the adjusted variables have the same categorizations as in Table 3).

## Discussion

Nearly all sexually active unimmunized adults are likely to be infected with HPV. To our knowledge, this is the first study to demonstrate a strong association of tobacco smoke exposure and HPV infection in adolescents by using biomarker measurement of serum cotinine levels. The present study showed that, in the weighted regression analysis, of the all the sociodemographic factors explored, only 3 were associated with HPV infections: history of sexual intercourse, tobacco smoke exposure, and age of the referent respondent. In this cross-sectional study, the significant dose-response relationship may suggest causality but is not necessarily proof. Using a national representative database, our study confirms the finding s of a couple of previous studies showing that tobacco smoke exposure is predictive of HPV infections among young adult women. However, biomarker measurements were not used in these studies. Kelsey et al [11] showed that tobacco smoke might potentiate HPV infection among younger adults (< 60 years) but not in older age group (60+ years). Sadate-Ngatchou et al showed that smoking women were more than twice likely to be infected with HPV than their non-smoking counterparts [12]. However, these studies did not use national representative data and did not use biomarkers to objectively determine tobacco smoke exposure. Our study is unique in that it also showed that even passive smoke exposure (serum cotinine levels 0.05–10 ng/mL) was also predictive of HPV infection as compared to unexposed women (cotinine levels <0.05ng/mL).

Our study also showed that there was a strong dose-response relationship between tobacco smoke exposure and HPV infections with the highest risk among active smokers and the lowest risk among the unexposed women. Even when the impact of tobacco smoke was explored by quartiles of cotinine levels there remained a very strong dose-response relationship in the univariate analyses and even after controlling for the various confounders. However, as stated above, this relationship is not necessarily causal because of the cross-sectional nature of the data collection procedures. This finding needs to be further explored.

HPV is now accepted as causally related to squamous cell carcinoma and other tumors of the ano-genital and head and neck regions [2]. The relationship between tobacco smoke exposure and HPV infection is not exactly clear. It is biologically plausible that tobacco smoke can actually cause increased HPV infection although the mechanisms have not been completely elucidated. Nicotine and many other toxic components of tobacco smoke have been demonstrated in the cervical mucus of patients who are exposed or are active smokers [13–15]. Alam et al. showed that benzo [a] pyrene, an important carcinogen in tobacco smoke detected in cervical mucus of women, enhances the persistence of HPV among women. [16].

The various toxins present in tobacco smoke may cause infection through the innate and humoral mechanisms [5]. As often happens in the respiratory tract, tobacco smoke may not only cause toxic injury to the epithelial cells but may actually increase production of adhesive molecules ultimately resulting in easy penetration of the virus into all epithelial cells to cause disease [17]. Smoking exposure also causes important humoral changes in the host. It has been shown to not only suppress the production of type-specific HPV antibodies [18, 19]. T helper 1 cells are known to stimulate B cells to produce type specific antibodies while NK cells can exert cytotoxic action on specific cells. In general, tobacco smoke inhibits T cell activity and NK cells [19].

Although, more studies are needed to determine whether tobacco smoke is causally related to increased HPV infections, the implication of this study is that sexually active young women may avoid HPV infections by avoiding tobacco smoke exposure, and of course, by being immunized with the HPV vaccine.

One limitation worth mentioning is that this was a cross-sectional study and therefore causality cannot necessarily be inferred. Another limitation is that the study was not able to capture younger adolescent in the 13–17 year old age group. However, the strength of this study is that that this was a national representative sample of 18 and 26 year old women in the United States and findings are generalizable to the US women in this age group. More studies are needed to underscore the dose-response relationship between tobacco smoke exposure and HPV infections.

## Summary, conclusion, and recommendations

Among young adult women 18 and 26 years of age, tobacco smoke exposure as measured by serum cotinine levels was associated with increased HPV infection with a significant dose response-relationship. Because HPV infections have been causally linked to certain oral and genital cancers and warts, this may be one more reason to warn young sexually active women to refrain from smoking. The findings of this study are worth further exploration to include younger adolescent in the 13 to 17 year age groups.

## Supporting information

S1 Data File to PloS (SAV)

## References

[1] Satterwhite CL, Torrone E, Meites E, Dunne EF, Mahajan R, Ocfemia MC, et al Sexually transmitted infections among US women and men: prevalence and incidence estimates, 2008. Sex Transm Dis. 2013 3;40(3):187–93. 10.1097/OLQ.0b013e318286bb53 Review. 23403598

[2] Dunne EF, Markowitz LE, Saraiya M, Stokley S, Middleman A, Unger ER, et al Centers for Disease Control and Prevention (CDC). CDC grand rounds: Reducing the burden of HPV-associated cancer and disease. MMWR Morb Mortal Wkly Rep. 2014 1 31;63(4):69–72. 24476977 PMC4584896

[3] Wiley D, Masongsong E. Human papillomavirus: the burden of infection. Obstet Gynecol Surv. 2006 6;61(6 Suppl 1):S3–14. Review. 10.1097/01.ogx.0000221010.82943.8c 16729902

[4] Winer RL, Hughes JP, Feng Q, Xi LF, Lee SK, O’Reilly SF, et al Prevalence and risk factors for oncogenic human papillomavirus infections in high-risk mid-adult women. Sex Transm Dis. 2012 11;39(11):848–56. 10.1097/OLQ.0b013e3182641f1c Erratum in: Sex Transm Dis. 2013 Nov;40(11):898. 23064533 PMC3476060

[5] Kum-Nji P, Meloy L, Herrod HG. Environmental tobacco smoke exposure: prevalence and mechanisms of causation of infections in children. Pediatrics. 2006 5;117(5):1745–54. 10.1542/peds.2005-1886 16651333

[6] Kelsey KT, Nelson HH, Kim S, Pawlita M, Langevin SM, Eliot M, et al Human papillomavirus serology and tobacco smoking in a community control group. BMC Infect Dis. 2015 1 9;15:8 10.1186/s12879-014-0737-3 25572638 PMC4296688

[7] Sadate-Ngatchou P, Carter JJ, Hawes SE, Feng Q, Lasof T, Stern JE, et al Determinants of High-Risk Human Papillomavirus Seroprevalence and DNA Prevalence in Mid-Adult Women. Sex Transm Dis. 2016 3;43(3):192–8. 10.1097/OLQ.0000000000000409 26859807 PMC4748390

[8] CDC. National Health And Nutrition Examination Survey: http://wwwn.cdc.gov/nchs/nhanes/search/nhanes09_10.aspx

[9] Watts RR, Langone JJ, Knight GJ, Lewtas J. Cotinine analytical workshopbreport: consideration of analytical methods for determining cotinine in human body fluids as a measure of passive exposure to tobacco smoke. Environ Health Perspect. 1990 3;84:173–82. 10.1289/ehp.9084173 2190812 PMC1567638

[10] Gravitt PE, Peyton CL, Alessi TQ, Wheeler CM, Coutlee F, Hildesheim A, et al Improved Amplification of Genital Human Papillomaviruses. J Clin Microbiol 2000; 38: 357–361. 10618116 10.1128/jcm.38.1.357-361.2000 PMC88724

[11] CDC. National Health And Nutrition Examination Survey: https://wwwn.cdc.gov/Nchs/Nhanes/2009-2010/HPVSWR_F.htm. (retrieved on 11/03/2016)

[12] IBM Corp. Released 2012. IBM SPSS Statistics for Windows, Version 21.0. Armonk, NY: IBM Corp.

[13] Prokopczyk B, Cox JE, Hoffmann D, Waggoner SE. Identification of tobacco-specific carcinogen in the cervical mucus of smokers and nonsmokers. J Natl Cancer Inst. 1997 6 18;89(12):868–73. 10.1093/jnci/89.12.868 9196253

[14] McCann MF, Irwin DE, Walton LA, Hulka BS, Morton JL, Axelrad CM. Nicotine and cotinine in the cervical mucus of smokers, passive smokers, and nonsmokers. Cancer Epidemiol Biomarkers Prev. 1992 Jan-Feb;1(2):125–9. 1306094

[15] Sasson IM, Haley NJ, Hoffmann D, Wynder EL, Hellberg D, Nilsson S. Cigarette smoking and neoplasia of the uterine cervix: smoke constituents in cervical mucus. N Engl J Med. 1985 1 31;312(5):315–6. 10.1056/nejm198501313120516 3965965

[16] Alam S, Conway MJ, Chen HS, Meyers C. The cigarette smoke carcinogen benzo[a]pyrene enhances human papillomavirus synthesis. J Virol. 2008 1;82(2):1053–8. Epub 2007 Nov 7. 10.1128/JVI.01813-07 . 17989183 PMC2224590

[17] Scott DA, Palmer RM. The influence of tobacco smoking on adhesion molecule profiles. Tob Induc Dis. 2002 1 15;1(1):7–25. 10.1186/1617-9625-1-1-7 19570245 PMC2671531

[18] Simen-Kapeu A, Kataja V, Yliskoski M, Syrjänen K, Dillner J, Koskela P, et al Smoking impairs human papillomavirus (HPV) type 16 and 18 capsids antibody response following natural HPV infection. Scand J Infect Dis. 2008;40(9):745–51. 10.1080/00365540801995360 19086247

[19] Kalra R, Singh SP, Savage SM, Finch GL, Sopori ML. Effects of cigarette smoke on immune response: chrnic exposure to cigarette smoke impairs antigen-mediated signaling in T cells and depletes IP3-sensitive Ca(2+) stores. J Pharmacol Exp Ther. 2000 4;293(1):166–71. 10734166

